# Hospital Meetings

**Published:** 1903-07-04

**Authors:** 


### HOSPITAL MEETINGS.

### CENTRAL LONDON THROAT AND EAR HOSPITAL.

The annual meeting of the committee and subscribers of this hospital was held on Wednesday, June 24th, Captain Jessel, M.P., being in the chair. After a sympathetic allusion to the loss the hospital had sustained during the past year through the death of Mr. Lennox Browne, the Chairman went on to say that the hospital was much to be congratulated on possessing a medical staff that was so self-sacrificing and able. The past year had been a record one in many respects. The total of in-patients admitted during 1902 was 339, which was the highest number in the history of the hospital. The number of new out-patients was 9,037, which was also the highest on record. Against this increase in the work of the hospital it was most gratifying to be able to set a decrease in the expenditure, which showed that the financial resources were carefully nursed. The Chairman then drew attention to the fact that 76 per cent. of the in-patients came from the provinces, and of the 146 who came from London, only 16 were from the West Central district where the hospital was situated. It was also to be noted, said Captain Jessel, that whereas the cost to the hospital of the in-patients from the provinces amounted to £575, the financial support contributed from the country only reached a total of £82, and the committee hoped in future to receive greater support from generous donors, especially in those districts from whence patients came. They had a great work before them—namely, the rebuilding of the in-patient department—which scheme had been strongly and repeatedly recommended by the Council of King Edward's Hospital Fund. Each year the official visitors of this Fund had inspected the hospital, and, while they had reported favourably on the work of all departments, they had one and all been deeply impressed with the limited space at the disposal of the hospital, and had expressed the opinion in no uncertain voice that it was quite insufficient for their large operative practice. The absence of a separate operating-room was also a very great defect. The probable cost for building and completely equipping a new building to accommodate 30 patients and the attendant staff would be £15,000. The new department would be erected on vacant land adjoining the hospital, which had been purchased by the committee some years ago. Towards the re-building fund they had already received £3,000, which included two donations from the King's Fund amounting to £800 in all. It was only by making a special appeal to the public that they could hope to raise so large a sum as they needed.

The Mayor of St. Pancras, who moved the adoption of the report, said he regretted to see that in spite of the good work done by the hospital, it was unable to pay its way, and was obliged to treat its legacies as income instead of capital.

At a later stage of the proceedings Dr. J. Dundas Grant proposed that the Mayor of St. Pancras and his successors should be *ex-officio* members of the committee, which proposition was agreed to by the present holder of that office. The ordinary business of the meeting was then transacted.

### FRIEDENHEIM HOSPITAL.

The annual meeting of Friedenheim Hospital was held on the 27th ult. in the Lecture Hall of the School for the Blind, Mr. John Langton in the chair. There was a full attendance.

After the singing of the hymn, "We have not known Thee as we ought," and opening prayers, the Chairman said the work of the hospital has been much blessed throughout the year. Following the building of the new home for the nurses, opened a year ago, electric light had been installed throughout the hospital; four beds for cancer and two for children had been added, and other improvements had been carried out. No less a sum than £1,500 was required to pay for these alterations.

Lord Kinnaird, in proposing the adoption of the report, accounts, and medical statement, alluded to the fact that 82 deaths had taken place during the year. But for this Home of Peace he thought these poor people's end would have been a dark and dreary one.

Mr. Baines and others testified to the necessity for and value of the work done by Miss Davidson and the nurses, speaking of the difficulties and responsibilities of tending the dying, and dealing with the religious aspect of the work.

Dr. A. T. Schofield emphasised the need for an adequate cancer ward for women; the six beds just provided had been filled immediately on completion of the alterations. Although the sum required to pay for improvements was estimated at £1,500, £1,000 would suffice if subscribed without delay.

Mr. J. H. Tritton, hon. treasurer, said he wished to call special attention to the collection of £71 17s. 9d. from St. Michael's, Chester Square, and to express the thanks of the subscribers, and others interested, to Canon Fleming for the sermons preached by him on behalf of the hospital.

The Benediction having been pronounced by Prebendary Webb-Peploe, the proceedings terminated, and the visitors adjourned to inspect the hospital.

The small ward for female cancer cases has been made out of what was formerly part of the accommodation for the nursing staff; while the extra room for two beds in the children's ward has been contrived by taking in a bathroom and passage. Both are prettily decorated, the one in white and green, the other in white and blue.

The cost of the Metropolitan Police during the year to March 31, 1903, was £2,141,859; £990,689 of this sum was borne by the rates, Kensington providing the largest amount.

Ninety pounds has been collected this year, on Hospital Saturday, in aid of the Ballymena Cottage Hospital. This total is the highest realised for this hospital from this source since 1893, when the amount collected was £93.

